# Supplementary material for: Enhancing Competencies and Professional Upskilling of Mobile Healthcare Unit Personnel at the Hellenic National Public Health Organization
Source: Healthcare (Basel). 2025 Jul 15;13(14):1706. doi: 10.3390/healthcare13141706 (PMC12296029; doi:10.3390/healthcare13141706)
Supplement: Supplementary file 1 [file healthcare-13-01706-s001.zip › healthcare-3667900-supplementary.pdf]

Supplementary data of the manuscript ""Enhancing Competencies and Professional Upskilling of Mobile Healthcare Unit Personnel at the Hellenic National Public Health Organization"

Table S1. Mapping of Training Modules to Core Public Health Competency Domains

The table below presents the alignment of training modules with core public health competency domains, based on the WHO-ASPHER Competency Framework for the Public Health Workforce in the European Region and the WHO Global Competency and Outcomes Framework for Universal Health Coverage.

Module Title / Thematic Area	Mapped Competency Domains (WHO/ASPHER-aligned)	Competency Examples
**I. Prevention – Health Promotion**	**- Health Promotion and Disease Prevention**<br>**- Epidemiological Surveillance**<br>**- Community Engagement**	**- Implement community-based screening and health promotion initiatives**<br>**- Interpret basic epidemiological data**
**Cancer prevention and screening**	Disease Prevention & Health Promotion	Design and promote screening initiatives (e.g., breast, cervical, colorectal cancers)
**Vaccination protocols**	Immunization & Public Health Emergency Preparedness	Ensure adherence to immunization schedules and educate on vaccine benefits
**Health behavior change (smoking cessation, physical activity)**	Health Literacy & Health Promotion	Apply behavior change communication techniques
**Infectious diseases control**	Communicable Disease Management	Identify and act on infection control priorities in the field
**II. Provision of Care**	**- Clinical Public Health Practice**<br>**- Interdisciplinary Collaboration**<br>**- Quality of Care**	**- Conduct chronic disease follow-up and biomarker interpretation**<br>**- Ensure adherence to treatment guidelines**
**Biomarker monitoring, diabetes, cardiovascular care**	Non-Communicable Diseases (NCDs) & Chronic Care	Apply risk stratification and evidence-based management
**Treatment adherence**	Health Services Delivery & Management	Use motivational interviewing and digital tools to support adherence
**Frailty, neurodegenerative and psychiatric conditions**	Geriatric and Mental Health Competencies	Recognize and refer vulnerable populations for specialized care
**Basic health assessment and documentation**	Essential Clinical Care	Perform and document standard clinical assessments
**III. Social Welfare & Solidarity Initiatives**	**- Social Determinants of Health**<br>**- Ethics and Equity**<br>**- Health Advocacy**	**- Address psychosocial needs and support equity in service delivery**
**Violence prevention and counseling**	Psychosocial Support & Community Care	Deliver trauma-informed support and referrals
**Blood and organ donation awareness**	Ethics and Cultural Competency	Promote voluntary donation with respect to ethical and cultural norms

**Equity in access & health literacy**	Social Justice, Equity & Inclusion	Engage underserved groups with tailored communication
**IV. Digital Health Skills**	**- Digital and eHealth Competency**<br>**- Data Literacy**<br>**- Health Information Systems**	**- Use digital tools for teleconsultation, documentation, and care coordination**
**Use of telemedicine and mobile health platforms**	eHealth & Digital Tools	Operate and troubleshoot digital tools for remote service delivery
**Vaccination records and digital documentation**	Health Information Management	Ensure secure and standardized digital record keeping
**Interdisciplinary digital collaboration**	Teamwork & Communication via ICT	Coordinate care and referrals using electronic systems

Table S2. Intra-Question Analysis of Knowledge Improvement

The observed results indicate a consistent improvement across all thematic areas following the intervention. The largest relative gains were observed in questions initially associated with low pre-intervention knowledge (e.g., Q5, Q14, Q23, Q26). These results support the effectiveness of the educational program in enhancing participants' knowledge, particularly in the areas of Health Promotion and Digital Health.

Question	Category	% Correct Before	95% CI Before	% Correct After	95% CI After	Δ%
**Q1**	Health promotion	0.585	(0.548, 0.621)	0.744	(0.709, 0.777)	0.160
**Q2**	Care provision	0.649	(0.612, 0.683)	0.656	(0.618, 0.692)	0.007
**Q3**	Health promotion	0.412	(0.376, 0.449)	0.575	(0.536, 0.613)	0.162
**Q4**	Social Welfare	0.901	(0.877, 0.921)	0.940	(0.918, 0.956)	0.038
**Q5**	Health promotion	0.284	(0.252, 0.319)	0.673	(0.635, 0.709)	0.389
**Q6**	Social Welfare	0.877	(0.850, 0.899)	0.924	(0.900, 0.942)	0.047
**Q7**	Digital Health	0.215	(0.186, 0.247)	0.378	(0.341, 0.416)	0.163
**Q8**	Digital Health	0.820	(0.790, 0.847)	0.919	(0.895, 0.938)	0.099
**Q9**	Care provision	0.682	(0.646, 0.716)	0.856	(0.826, 0.881)	0.173
**Q10**	Digital Health	0.433	(0.396, 0.470)	0.724	(0.688, 0.757)	0.291
**Q11**	Care provision	0.772	(0.738, 0.803)	0.916	(0.888, 0.938)	0.144
**Q12**	Health promotion	0.235	(0.203, 0.270)	0.242	(0.208, 0.280)	0.007

**Q13**	Digital Health	0.671	(0.634, 0.706)	0.864	(0.832, 0.890)	0.193
**Q14**	Health promotion	0.370	(0.334, 0.407)	0.705	(0.667, 0.741)	0.335
**Q15**	Care provision	0.759	(0.724, 0.790)	0.765	(0.728, 0.798)	0.006
**Q16**	Care provision	0.437	(0.399, 0.475)	0.618	(0.578, 0.656)	0.181
**Q17**	Care provision	0.219	(0.188, 0.253)	0.422	(0.384, 0.461)	0.203
**Q18**	Care provision	0.573	(0.535, 0.610)	0.740	(0.700, 0.776)	0.167
**Q19**	Health promotion	0.917	(0.892, 0.937)	0.956	(0.933, 0.972)	0.039
**Q20**	Health promotion	0.763	(0.727, 0.796)	0.829	(0.793, 0.860)	0.066
**Q21**	Health promotion	0.588	(0.550, 0.624)	0.859	(0.823, 0.888)	0.271
**Q22**	Health promotion	0.030	(0.020, 0.045)	0.101	(0.077, 0.132)	0.071
**Q23**	Health promotion	0.161	(0.135, 0.191)	0.480	(0.438, 0.522)	0.319
**Q24**	Social Welfare	0.807	(0.774, 0.836)	0.878	(0.845, 0.905)	0.071
**Q25**	Social Welfare	0.520	(0.481, 0.558)	0.791	(0.752, 0.826)	0.271
**Q26**	Social Welfare	0.287	(0.253, 0.324)	0.629	(0.588, 0.668)	0.342
**Q27**	Health promotion	0.636	(0.598, 0.672)	0.832	(0.796, 0.864)	0.196
**Q28**	Health promotion	0.610	(0.572, 0.647)	0.797	(0.758, 0.831)	0.187
**Q29**	Care provision	0.264	(0.232, 0.300)	0.513	(0.472, 0.554)	0.249
**Q30**	Care provision	0.386	(0.350, 0.423)	0.639	(0.598, 0.678)	0.253
